# Serum Levels of the Adipokine Zinc-**α**2-glycoprotein Are Decreased in Patients with Hypertension

**DOI:** 10.1155/2014/374090

**Published:** 2014-02-09

**Authors:** Hui Juan Zhu, Xiang qing Wang, Hui Pan, Feng ying Gong, Dian xi Zhang, Nai shi Li, Lin jie Wang, Hong bo Yang

**Affiliations:** Department of Endocrinology, Key Laboratory of Endocrinology of Ministry of Health, The Translational Medicine Center of PUMCH, Peking Union Medical College Hospital, Chinese Academy of Medical Sciences and Peking Union Medical College, Beijing 100730, China

## Abstract

*Objective*. Zinc-*α*2-glycoprotein (ZAG) has recently been proposed as a new adipokine involved in body weight regulation. The purpose of this study is to investigate serum levels of ZAG in patients with hypertension and its association with related characteristics. *Methods*. 32 hypertension patients and 42 normal controls were recruited and the relationship between serum ZAG, total and high molecular weight (HMW) adiponectin, and tumor necrosis factor-*α* (TNF*α*) determined by enzyme-linked immunosorbent assay (ELISA) and metabolic-related parameters was investigated. *Results*. Serum ZAG concentrations were significantly lowered in patients with hypertension compared with healthy controls (61.4 ± 32 versus 78.3 ± 42 *μ*g/mL, *P* < 0.05). The further statistical analysis demonstrated that serum ZAG levels were negatively correlated with waist-to-hip ratio (WHR) (*r* = −0.241, *P* < 0.05) and alanine aminotransferase (ALT) (*r* = −0.243, *P* < 0.05). Additionally, serum HMW adiponectin significantly decreased, while TNF*α* greatly increased in hypertension patients as compared with healthy controls (2.32 ± 0.41 versus 5.24 ± 1.02 *μ*g/mL, 3.30 ± 1.56 versus 2.34 ± 0.99 pg/mL, *P* < 0.05). *Conclusions*. Serum ZAG levels are significantly lowered in hypertension patients and negatively correlated with obesity-related item WHR, suggesting ZAG is a factor associated with hypertension.

## 1. Introduction

Obesity is now a major health problem as it predisposes to insulin resistance, type 2 diabetes, cardiovascular malfunction, and cancer. Although the pathogenesis of obesity and its associated comorbidities are multifactorial, growing evidence suggests that altered production of adipose-derived protein factors (adipokines), such as leptin, tumour necrosis factor *α* (TNF*α*), adiponectin, and chemerin, plays an important role. For example, adipokines induce the functional and structural changes in the vessels by endothelial dysfunction, vascular smooth muscle cell (VSMC) proliferation and migration, and vascular inflammation, thereby regulating vascular responses to constrictor and dilator stimuli and contributing to the increased arterial pressure [1].

Zinc-*α*2-glycoprotein (ZAG, also called AZGP1) is a newly identified adipokine. Recent work from both our group and others has demonstrated ZAG levels in the serum and adipose tissue of obese patients and obese mice are significantly lower relative to subjects and mice of normal weights [2–4]. ZAG levels are negatively correlated with body weight and body fat mass [2, 5, 6]. The administration of ZAG in mice dramatically diminishes body weight and fat mass of normal, *ob/ob*, and high-fat-diet- (HFD-) induced obese mice by regulating lipogenesis and lipolysis [2, 7, 8]. The rs4215 (A/G) single nucleotide polymorphism (SNP) in the ZAG gene is associated with obesity in the Chinese North Han population [9]. In addition to the close link between ZAG levels and obesity, very recent work performed by Stepan et al. [10], Leal et al. [11], and Tedeschi et al. [12] also demonstrates that serum ZAG levels are increased in patients with preeclampsia, hemodialysis (HD), or advanced heart failure patients compared with response controls. These results indicate that ZAG is a novel lipid-mobilizing adipokine which is associated with obesity and some components of its related complication including diabetes and heart failure.

But, to date, no study has quantified circulating ZAG levels in patients with hypertension. It has not been evaluated so far whether serum levels of ZAG are changed in hypertension patients in a similar way to other adipokines and might, therefore, contribute to the pathogenesis of hypertension. So, in the present study, we investigate serum levels of ZAG in patients with or without hypertension and its association with related clinical and biochemical characteristics.

## 2. Subjects and Methods

### 2.1. Study Participants: Clinical and Biochemical Characterization

Thirty-two newly diagnosed high blood pressure (HBP) patients and forty-two healthy control subjects were recruited from outpatient in Peking Union Medical College Hospital, and all subjects underwent physical examination, anthropometric measurements, and biochemical screening. The inclusion criteria of HBP patients are as follows: (1) systolic blood pressure (SBP)≧140 mmHg or diastolic blood pressure (DBP)≧90 mmHg without any antihypertension treatment; (2) normal liver function (ALT (U/L) < 40, AST (U/L) < 40) and renal function (Cr (umol/L) < 84, BUN (mmol/L) < 7.14); (3) age range from 25 y to 70 y. Patients with chronic disease history and endocrine diseases were excluded. Blood pressure (BP) was measured using a mercury sphygmomanometer (Baumanometer, WA Baum) and standard-sized cuffs (12 to 14 cm wide) according to a standardized protocol. The subjects had a rest at least 10 minutes before BP measurement. The values of SBP and DBP were taken as the mean of 2 readings with 2- to 3-minute interval on the right arm of the subjects. Body weight, height, and percentage of body fat (fat%) of every subject in light clothing without socks and shoes were obtained by bioelectrical impedance analyzer (TANITA, TBF-215, Japan). Body mass index (BMI) was calculated as weight divided by squared height, and BMI of all subjects ranged from 18.0 to 25.0 kg/m^2^. Waist circumference was measured with a soft tape on standing subjects midway between the lowest rib and the iliac crest. Hip circumference was measured over the widest part of the gluteal region. Waist-to-hip ratio (WHR) was calculated after waist and hip circumferences were measured. All blood samples were collected from an antecubital vein between 8.00 and 9.00 a.m. following an overnight fasting. Total cholesterol (TC), triglycerides (TG), high-density lipoprotein cholesterol (HDL-c), low-density lipoprotein cholesterol (LDL-c), fasting blood glucose (FBG), creatinine (Cr), uric acid (UA), aspartate aminotransferase (AST), alanine aminotransferase (ALT), and blood urea nitrogen (BUN) were measured by routine automated laboratory methods. Patients with chronic disease history, endocrine diseases, and any other disease which might influence our results were excluded. The study was approved by the ethics committee of Peking Union Medical College Hospital. All participants gave written informed consent before taking part in the study.

### 2.2. Serum Adipokines Assay

Blood samples were centrifuged at 3000 g for 10 min and serum aliquots were stored at −80°C until analysis. Serum ZAG level was determined by commercially available human zinc-alpha2-glycoprotein ELISA kit (Biovendor, laboratorni medicina, Czech Republic) according to the manufactures instruction. The minimum detection dose is 0.673 ng/mL. The serum levels of total adiponectin, high molecular weight (HMW) adiponectin, and TNF*α* were also determined by commercially available human ELISA kits (Adipo Bioscience, Inc., USA, for total adiponectin, and R&D Systems Quantikine ELISA, USA, for high molecular weight (HMW) adiponectin and TNF*α*). The minimum detection dose is 31 pg/mL for total adiponectin, 0.195 ng/mL for HMW adiponectin, and 1.6 pg/mL for TNF*α*. All antibodies used in these assays were specific for human targeted adipokines with no detectable cross-reactivities to other adipokines and genus. All samples were assayed in duplicate and random order, and lab measurements were performed blinded to the readings of blood pressure. The intra-assay CVs were 6.2% for ZAG, 2.4% for adiponectin, 6.8% for HMW adiponectin, and 1.8% for TNF*α*, respectively.

### 2.3. Data Analysis

The Shapiro-Wilk normality test was used for all continuous variables to characterize the data distribution. Results were expressed as mean ± standard deviation (SD). Skewed data were log-transformed and Mann-Whitney *U* test was used if data were still not normally distributed for further analysis. Student's *t*-test was used to evaluate differences between the two study groups in normally distributed continuous variables. Partial correlations among the study variables were tested controlling for age, gender, and BMI on SPSS 13.0 for Windows (SPSS Inc, Chicago, IL, USA); *P* < 0.05 was considered as statistically significant in all analyses.

## 3. Results

### 3.1. General Characteristics

The differences of anthropometric and laboratory measurements in healthy controls and HBP patients are displayed in Table 1. As expected, HBP patients had higher SBP (*P* < 0.001) and DBP (*P* < 0.001) than the normal group in addition to their higher FBG (*P* = 0.006), TC (*P* = 0.025), LDL-c (*P* = 0.014), waist (only for female) (*P* < 0.001), and WHR (*P* < 0.001). However, there was no significant difference with regard to age, height, weight, BMI, hips, fat%, ALT, AST, Cr, BUN, UA, TG, and HDL-c in these two groups.

### 3.2. Serum Levels of ZAG and Other Adipokines in HBP Patients and Healthy Controls

Serum levels of ZAG, total adiponectin, HMW adiponectin, and TNF*α* in healthy controls and HBP patients were determined by commercial available ELISA kits. As shown in Table 2, HBP patients presented 21.6% lower levels of serum ZAG when compared with healthy controls (61.4 ± 32 versus 78.3 ± 42 *μ*g/mL, *P* < 0.05). Like ZAG, serum HMW adiponectin also significantly decreased in HBP patients (*P* < 0.05). However, serum TNF*α* levels in HBP patients increased by 41% as compared with healthy controls (*P* = 0.002). No significant difference was found in serum total adiponectin level between HBP patients and healthy controls (*P* = 0.304, Table 2).

### 3.3. Correlations of Serum Levels of Adipokines with Anthropometric and Biochemical Items in HBP Patients and Healthy Controls

**Figure 1:**
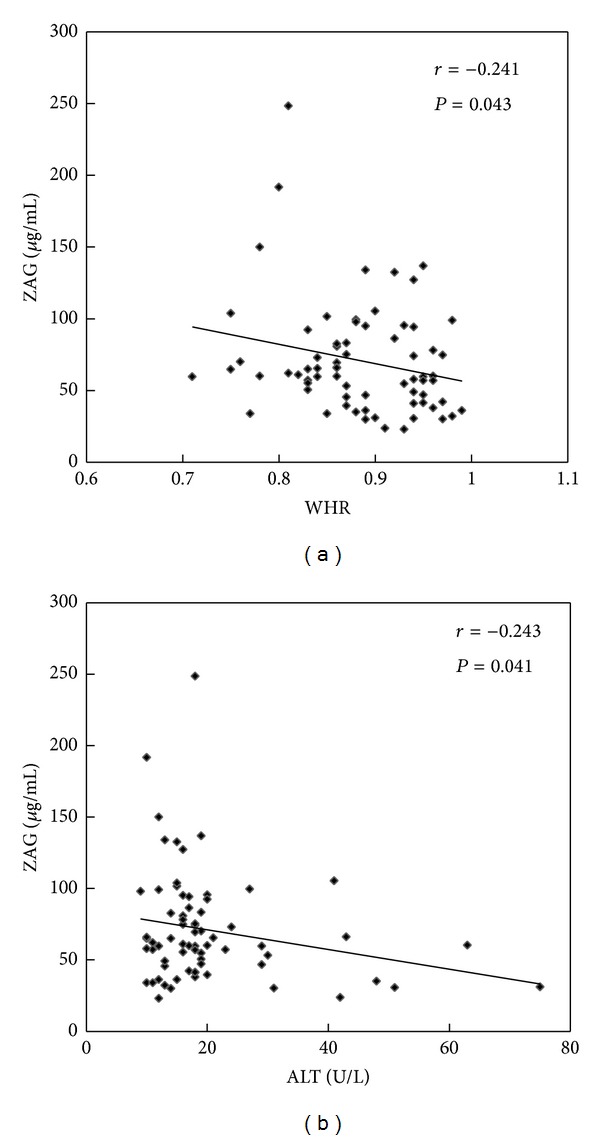
Correlation analysis of serum ZAG levels and WHR (a) and ALT (b) in healthy controls and HBP patients.

**Figure 2:**
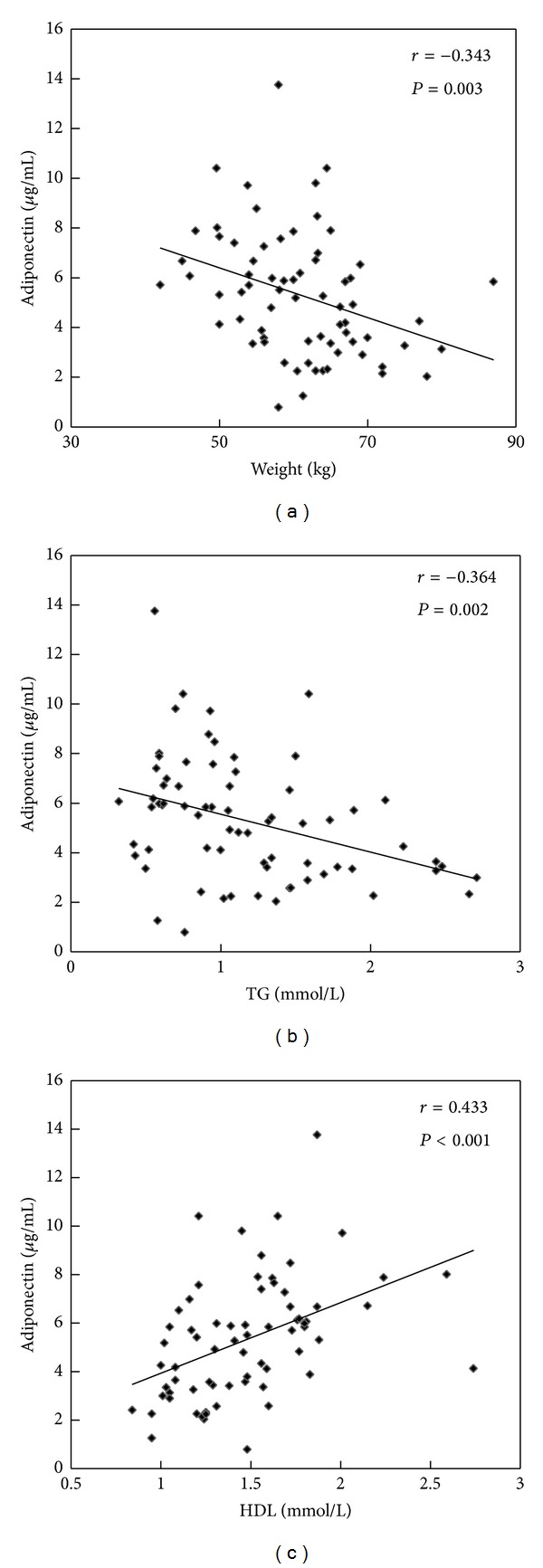
Correlation analysis of serum adiponectin levels and weight (a), TG (b), HDL (c) in healthy controls and HBP patients.

**Figure 3:**
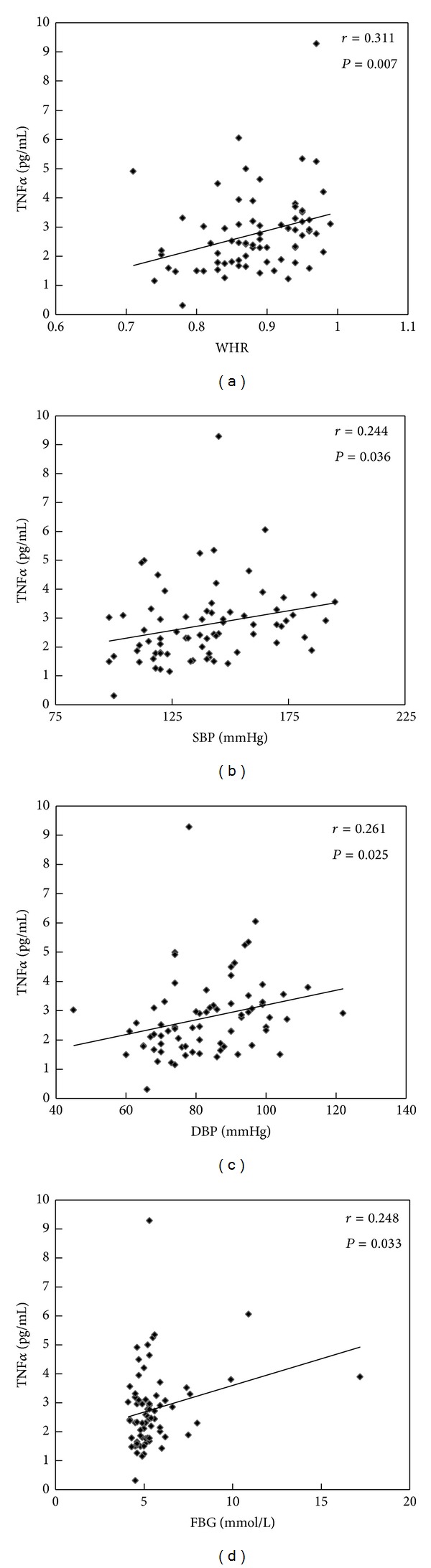
Correlation analysis of serum TNF*α* levels and WHR (a), SBP (b), DBP (c), and FBG (d) in healthy controls and HBP patients.

Pearson or spearman correlation coefficient was calculated to analyze the relationship between ZAG, adiponectin, TNF*α*, and anthropometric and biochemical items in HBP patients and healthy controls. As depicted in Figure 1, serum ZAG levels were negatively correlated with WHR and ALT, respectively, in all subjects (*r* = −0.241, *P* < 0.05; *r* = −0.243, *P* < 0.05) (Figure 1).  Figure 2 indicates that serum adiponectin levels were inversely correlated with body weight (*r* = −0.343, *P* < 0.05) and TG, respectively, (*r* = −0.364, *P* < 0.05), but were positively correlated with HDL (*r* = 0.433, *P* < 0.05). Unlike ZAG and adiponectin, serum TNF*α* levels were positively correlated with WHR (*r* = 0.311, *P* < 0.05), SBP (*r* = 0.244, *P* < 0.05), DBP (*r* = 0.244, *P* < 0.05), and FBG (*r* = 0.248, *P* < 0.05) in all subjects (Figure 3).

## 4. Discussion

ZAG is a newly identified adipokine secreted by adipocytes. Recent work from both our group and others has demonstrated that ZAG is closely linked to obesity and obesity-related metabolic disease and has been proposed as a candidate factor in the regulation of body weight and blood glucose [3, 13, 14]. In the current study, we show for the first time that median serum ZAG levels are 21.6% lower in HBP patients as compared with healthy controls. As we all know, hypertension is an important component of metabolism syndrome, which has been proved to associate with many adipokines including adiponectin, leptin, angiotensin, perivascular relaxation factors, and resistin [15–18]. These adipokines keep the vascular homeostasis by acting on vascular component cells including endothelial cells and macrophages. In this study, we found that ZAG may be another adipokine involved in blood pressure control. In contrast to our finding, Stepan et al. [10] found maternal serum ZAG concentrations are significantly increased in preeclampsia which is characterized by hypertension, proteinuria, and endothelial dysfunction. The different change trend of serum ZAG level in preeclampsia and HBP patients may be explained by the different pathogenesis of these two kinds of diseases. Additionally, studies performed by Stepan et al. [10] and Philipp et al. [19] indicated that renal elimination was a major route by which physiologic ZAG serum levels are maintained, and renal function was an independent predictor of circulating ZAG because serum ZAG levels were almost 2-fold higher in chronic hemodialysis patients as compared with controls. The subjects recruited in our study including HBP patients and healthy controls have normal renal function shown as normal creatinine and uric acid in Table 1, suggesting that the lower serum ZAG levels in HBP patients are indeed associated with blood pressure but not renal function.

Further correlation analysis showed that serum ZAG levels were negatively related with WHR and ALT in all subjects although ALT of these subjects was in normal range. The finding further confirms that ZAG is an important factor which is closely linked to obesity and obesity-related metabolic disease. Yilmaz et al. [20] found that serum ZAG concentrations were the only independent predictor of the number of metabolic syndrome components in patients with nonalcoholic fatty liver disease (NAFLD) after stepwise linear regression analysis. Leal et al. [11] demonstrated that ZAG levels were inversely associated with TNF*α* and vascular cell adhesion molecule-1 (VCAM-1) but positively correlated with anti-LDL(−) autoantibodies in HD patients. Serum ZAG levels have also been proved to increase in advanced heart failure patients compared with age-matched healthy controls [12]. All of these findings demonstrate that ZAG plays an important role in the pathogenesis of metabolic disease and would be a promising target for antiobesity and antimetabolic syndrome therapies.

Yeung et al. reported that ZAG levels in male were significantly higher than those in female in Southern Chinese subjects [21]. In our present study, we found that there was a trend that ZAG levels of man were higher than those of women in both healthy controls (88.3 ± 54.2 versus 71.7 ± 36.0 *μ*g/mL, *P* = 0.226) and HBP patients (65.3 ± 35.6 versus 53.0 ± 23.3 *μ*g/mL, *P* = 0.329). However, the gender differences were not statistically significant. The reason of this result may be the relative small sample size. Therefore, a further investigation in a large sample is required to elucidate this question.

Adiponectin is another adipokine, which displays a variety of protective actions in obesity-induced pathological conditions, including hypertension, diabetes mellitus, hepatic steatosis, atherosclerosis, and ischemic heart disease [22–25]. In our present study, we found that serum HMW adiponectin levels were lower in the HBP patients and serum total adiponectin levels were inversely correlated with body weight and TG, respectively, but were positively correlated with HDL. Similar results were obtained by Adamczak et al. in 2003 who demonstrated that adiponectin levels were significantly lower in patients with essential hypertension compared with those in BMI-matched normotensive controls, and an inverse correlation was observed between adiponectin concentration and mean blood pressure [26]. In addition, Choi et al. proved that adiponectin levels are negatively associated with blood pressure in patients with type 2 diabetes and metabolic syndrome [27].

Previous studies reported that HMW adiponectin may be the active form of adiponectin, and it is recognized as a strong predictor of metabolic disease and its complications [28–31]. Epidemiological studies also showed that HMW adiponectin was correlated better with glucose tolerance and may be a better marker for coronary artery disease and obesity than total serum adiponectin [30, 32, 33]. In agreement with these results, we found the levels of HMW but not total adiponectin decreased in the patients with HBP. Importantly, recent studies by Gao et al. and others revealed a positive association between ZAG mRNA and adiponectin mRNA levels in both visceral and subcutaneous fat of human subjects [34–36]. The levels of ZAG released by human adipocytes have been demonstrated to be comparable with that of adiponectin [35]. The parallel expression pattern for these two adipokines may suggest that ZAG could act like adiponectin in protecting against the obese state. Similar phenomenon was observed in our present study showing both serum ZAG and HMW adiponectin levels are notably declined in HBP patients in comparison with healthy controls. In light of the beneficial effects of ZAG and adiponectin in decreasing body fat and improving insulin sensitivity, both adipokines have been suggested to be therapeutic targets of antiobesity and antidiabetes therapeutics.

Unlike ZAG and adiponectin, TNF*α* levels in the present study were higher in the HBP patients as compared with healthy controls and positively correlated with WHR, SBP, DBP, and FBG. Inflammation has been considered a key component in the pathogenesis of hypertension which is a low-grade inflammatory condition induced by TNF*α*. TNF*α*, which is an inflammation promoter, may play an important role in the modulation of hypertensive response. Several studies *in vitro* and *in vivo* studies suggest the existence of cross-talk between TNF*α* and the renin-angiotensin system (RAS), which is an important part of the pathogenesis of hypertension [37, 38]. Bogdański et al. found that the serum TNF*α* levels in essential hypertension (EH) patients were much higher than in normotensive individuals, which is in agreement with our results [39]. Recently, Li [40] demonstrated a significant association of TNF*α* G308A gene polymorphism with EH, and the A allele carriers in G308A SNP site have increased susceptibility to EH compared with the G allele carriers.

Although the factors that downregulated ZAG are not well understood, obesity-related inflammation could be involved in the ZAG regulation. Gao et al. [36] showed that chronic treatment with TNF*α* led to a significant decrease in ZAG expression and secretion in adipocytes. Mracek et al. [2] and Bao et al. [41] also found a chronic and dose-dependent decrease in ZAG mRNA levels after treatment with TNF*α*. Inhibition of ZAG expression and release by TNF*α* may increase the susceptibility to lipid accumulation in adipose tissue and liver in obesity state [2]. Moreover, the parallel expression pattern of ZAG and adiponectin, similarly regulated by TNF*α* [41], suggests that ZAG could act like adiponectin in protecting against the obese state [42].

## 5. Limitations

Our study firstly reported the lower serum ZAG level in HBP patients in comparison with healthy controls in Chinese North Han subjects. This primary result needs to be confirmed in the larger samples and other races. Further efforts should also be undertaken in molecular and cell levels to identify the biological function of ZAG in occurrence and development of hypertension.

## 6. Conclusions

In summary, serum ZAG and HMW adiponectin levels are significantly lowered, while TNF*α* greatly increased in hypertension patients. Serum ZAG levels were negatively correlated with WHR. These findings implies that ZAG may be an important factor and is associated with hypertension. Prospective studies are needed to better demonstrate the exact role of ZAG in hypertension.

## Figures and Tables

**Table 1:** General clinical characteristics of healthy controls and HBP patients.

	Healthy controls	HBP patients	*P* value
M/F (*n*)	17/25 (42)	22/10 (32)	**0.016**
Age (y)	53.1 ± 8.0	56.9 ± 13.4	0.160
Height (cm)	164.9 ± 9.2	162.0 ± 7.3	0.145
Weight (kg)	62.5 ± 10.0	59.5 ± 6.3	0.136
BMI (kg/m^2^)	22.9 ± 2.0	22.6 ± 1.5	0.610
Waist (cm)	78.5 ± 8.1	87.1 ± 5.0	**<0.001**
Male	83.5 ± 6.3	86.9 ± 4.7	0.063
Female	75.0 ± 7.4	87.7 ± 5.7	**<0.001**
Hips (cm)	93.5 ± 5.2	93.6 ± 4.2	0.726
Male	95.5 ± 4.6	92.8 ± 4.1	0.069
Female	91.6 ± 5.1	95.2 ± 4.1	0.057
WHR	0.84 ± 0.06	0.93 ± 0.04	**<0.001**
Male	0.87 ± 0.04	0.93 ± 0.03	**<0.001**
Female	0.82 ± 0.05	0.92 ± 0.05	**<0.001**
Fat (%)	26.6 ± 6.1	24.3 ± 6.7	0.126
SBP (mmHg)	122.9 ± 14.1	159.5 ± 17.6	**<0.001**
Male	128.4 ± 14.2	158.8 ± 18.1	**<0.001**
Female	119.1 ± 13.0	161.1 ± 17.1	**<0.001**
DBP (mmHg)	75.5 ± 11.1	92.3 ± 11.3	**<0.001**
Male	80.6 ± 12.2	92.0 ± 10.6	**0.004**
Female	72.0 ± 8.8	92.8 ± 13.3	**0.001**
FBG (mmol/L)	4.93 ± 0.65	6.24 ± 2.5	**0.006**
Male	4.99 ± 0.89	6.07 ± 1.71	**0.016**
Female	4.89 ± 0.43	6.63 ± 3.72	**0.025**
ALT (U/L)	21.4 ± 12.8	20.0 ± 12.8	0.643
AST (U/L)	22.3 ± 9.4	22.5 ± 9.7	0.944
Cr (umol/L)	78.7 ± 16.0	81.8 ± 13.4	0.382
BUN (mmol/L)	7.01 ± 11.2	5.32 ± 1.36	0.396
UA (mmol/L)	239.1 ± 92.7	258.7 ± 79.7	0.343
TC (mmol/L)	4.83 ± 0.78	5.25 ± 0.80	**0.025**
Male	4.80 ± 0.52	5.12 ± 0.81	**0.138**
Female	4.85 ± 0.92	5.55 ± 0.78	**0.038**
TG (mmol/L)	1.25 ± 0.58	1.08 ± 0.57	0.198
HDL-c (mmol/L)	1.38 ± 0.42	1.53 ± 0.39	0.138
LDL-c (mmol/L)	2.93 ± 0.68	3.32 ± 0.64	**0.014**
Male	2.85 ± 0.86	3.29 ± 0.66	**0.077**
Female	2.99 ± 0.53	3.40 ± 0.61	**0.038**

BMI: body mass index; WHR: waist-to-hip ratio; SBP: systolic blood pressure; DBP: diastolic blood pressure; FBG: fasting blood glucose; ALT: alanine aminotransferase; AST: aspartate aminotransferase; Cr: creatinine; BUN: blood urea nitrogen; UA: uric acid; TC: total cholesterol; TG: triglycerides; HDL-c: high-density lipoprotein cholesterol; LDL-c: low-density lipoprotein cholesterol.

**Table 2:** Serum levels of ZAG and other adipokines in HBP patients and healthy controls.

	Healthy controls	HBP patients	*P* value
ZAG (*μ*g/mL)	78.3 ± 42.0	61.4 ± 32.2	**0.020**
Male	88.3 ± 54.2	65.3 ± 35.6	0.113
Female	71.1 ± 31.0	53.0 ± 23.3	**0.029**
Total adiponectin (*μ*g/mL)	5.02 ± 2.23	5.61 ± 2.74	0.304
HMW adiponectin (*μ*g/mL)	5.24 ± 1.02	2.32 ± 0.41	**0.046**
Male	5.32 ± 1.66	2.31 ± 0.39	**0.004**
Female	5.83 ± 2.43	4.01 ± 3.07	0.484
TNF*α* (pg/mL)	2.34 ± 0.99	3.30 ± 1.56	**0.002**
Male	2.87 ± 0.95	3.11 ± 1.17	0.484
Female	1.98 ± 0.86	3.72 ± 2.20	**0.002**

ZAG: zinc-alpha2-glycoprotein; HMW adiponectin: high molecular weight adiponectin; TNF*α*: tumor necrosis factor *α*.

## Abbreviations

ZAG: Zinc-*α*2-glycoprotein
HMW adiponectin: High molecular weight adiponectin
TNF*α*: Tumor necrosis factor-*α*.
